# 3D biomimetic environment enabling ex utero trophoblast invasion and co-culture of embryos and somatic cells

**DOI:** 10.1016/j.xpro.2023.102456

**Published:** 2023-07-28

**Authors:** Niraimathi Govindasamy, Hongyan Long, Adrian Ranga, Britta Trappmann, Ivan Bedzhov

**Affiliations:** 1 Embryonic Self-Organization Research Group, Max Planck Institute for Molecular Biomedicine, Röntgenstraße 20, 48149 Munster, Germany; 2 Bioactive Materials Laboratory, Max Planck Institute for Molecular Biomedicine, Rontgenstraße 20, 48149 Munster, Germany; 3 Laboratory of Bioengineering and Morphogenesis, Department of Mechanical Engineering, KU Leuven, Leuven, Belgium

## Abstract

The first direct contact between the embryo and the mother is established during implantation. This process is inaccessible for direct studies as the implanting embryo is concealed by the maternal tissues. Here, we present a protocol for establishing a 3D biomimetic environment based on synthetic hydrogels which harbor key biomechanical properties of the uterine stroma. We describe steps for isolating and culturing embryos in PEG/DexMA hydrogel. We then detail the co-culture of embryos and endothelial cells in a microfluidic device.

For complete details on the use and execution of this protocol, please refer to Govindasamy et al. (2021)^1^ and Ozguldez et al. (2023).^2^

## Graphical abstract

**Figure:**
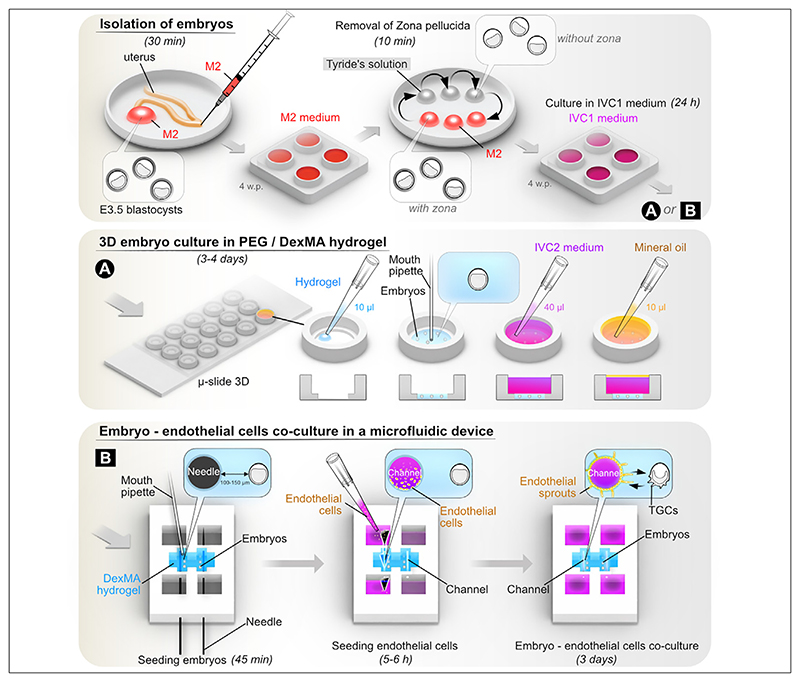

## Before You Begin

Investigating the process of implantation is technically challenging because of the intrauterine development in mammals. Hence, while almost half of the miscarriages in human-assisted reproduction are associated with implantation defects,^3,4^ the cellular mechanisms of this process are still insufficiently understood. In mice, implantation is initiated as the mural trophectoderm (TE) of the late blastocyst adheres to the uterine wall and differentiates into trophoblast giant cells (TGCs), which invade the endometrial stroma. At the same time, the stromal cells rapidly proliferate forming the decidua, a specialized compartment which engulfs the embryo to support its further development.^5^

To directly study the process of embryo invasion ex utero, we recently established a biomimetic environment based on synthetic hydrogels harboring key features of the decidualized stroma in mice.^1^ Here we provide a step-by-step protocol describing the rationale behind the design of the substrates, the biochemical assembly of the hydrogels and the embryo culture procedure.

The synthetic environment based on methacrylated dextran (DexMA) or polyethylene glycol (PEG) captures three key features of the uterine stroma, namely degradability, stiffness and adhesion. As the TGCs produce matrix metalloproteinases (MMPs) that promote embryo invasion,^6^ we incorporated MMP-cleavable peptide sequences, crosslinking the DexMA or PEG macromeres to establish degradable hydrogels. We adjusted the concentration of PEG and tuned the amount of the MMP-cleavable crosslinker of the DexMA hydrogels to achieve the decidua-like stiffness of the substrates. In addition, we incorporated RGD peptide sequences enabling integrin adhesion, which is essential for the invasion of the trophoblast.^1^ Thus, the designs of both PEG and DexMA hydrogels are based on the same principles, resembling the biomechanical properties of the murine uterine stroma.

This platform also supports the co-culture of embryos and somatic cells, thus providing a model system for studying embryo-maternal interactions. As an example, here we describe a setup for co-culturing embryos and endothelial cells. In the future, the 3D culture system can be further modified by incorporating other cell types of the maternal environment. Moreover, as the building materials are processable into hydrogels with controlled stiffness, cell-adhesive and degradative properties, the substrates can be tuned to mimic the biomechanical properties of the endometrial stroma in other mammalian species and molded into structures that resemble the architecture of the uterine tissues.

The culture system enables direct analysis of trophoblast invasion and modeling of the embryo-maternal interactions at the implantation site. In rodent embryos, the mural TE initiates the process of embryo invasion which is faithfully recapitulated in the 3D environment.^1^ In contrast, primate embryos, including humans, invade the uterine wall with the polar region of the TE.^5^ This feature of the implantation of the primate embryos is likely to be recapitulated in the 3D culture system. Potentially, stem cell-based embryo-like structures (embryoids, blastoids) could also be cultured in this environment. As such, this setup may provide a virtually unlimited amount of material for down-stream analysis. In addition, the co-culturing of embryos with somatic cells representing uterine tissues can serve as a platform to examine the “hidden” dynamics of the embryo-maternal crosstalk. In perspective, the characteristics of this biomimetic platform, based on the decidua-like stiffness, integrin adhesion, degradability and molding of the synthetic substrates, can serve as a foundation for the future development of more complex embryo culture environments.

### Institutional permissions

Animal experiments and husbandry were performed according to the German Animal Welfare guidelines and approved by the Landesamt für Natur, Umwelt und Verbraucherschutz Nordrhein-Westfalen (State Agency for Nature, Environment and Consumer Protection of North Rhine-Westphalia). Experiments that involve experimental animals must be conducted in compliance with all relevant institutional and governmental regulations. The relevant permissions from the institutional committees must be obtained before proceeding with the protocol.

## Key Resources Table

**Table:** 

Reagent Or Resource	Source	Identifier
Antibodies		
Oct4A (D6C8T) (1:200 dilution)	Cell Signaling	83932S RRID: AB_2721046
Troma1 (1:200 dilution)	Home-made (kind gift from Prof. Rolf Kemler)	N/A
Alexa 594 Donkey Anti-Rabbit (1:200 dilution)	Thermo Fisher Scientific	A-21207; RRID: AB_141637
Alexa 647 Donkey Anti-Rat (1:200 dilution)	Thermo Fisher Scientific	A-21247; RRID: AB_141778
Chemicals, peptides, and recombinant proteins		
Pregnant mare’s serum gonadotropin (PMSG)	Pregmagon	G62012B
Human chorionic gonadotropin (HCG)	Ovogest	04370
M2 medium	Sigma-Aldrich	M7167
Tyrode’s solution	Sigma-Aldrich	T1788
Mineral oil, suitable for mouse embryo culture	Merck	ES-005-C
DMEM F-12	Invitrogen	21331-046
DMEM powder without sodium bicarbonate	Thermo Fisher Scientific	12800017
Fetal calf serum (FCS)	Invitrogen	10828028
Penicillin/Streptomycin (Pen/Strep)	Sigma-Aldrich	P4333
L-Glutamine	Sigma-Aldrich	G7513
ITS-X	Invitrogen	51500-056
β-Estradiol	Sigma-Aldrich	E8875
Progesterone	Sigma-Aldrich	P0130
N-acetyl-L-cysteine (NAC)	Sigma-Aldrich	A7250
Sodium bicarbonate	Sigma-Aldrich	S5761
KSR	Invitrogen	10828028
DMSO	VWR	41640-100ML-D
DMEM high glucose	Sigma-Aldrich	D5671
Sodium pyruvate	Sigma-Aldrich	S8636
NEAA	Sigma-Aldrich	M7145
β-Mercaptoethanol (99%)	Sigma-Aldrich	M3148
Gelatin solution	Sigma-Aldrich	G1393
Trypsin (0.25%)	Invitrogen	25200056
PBS	Sigma-Aldrich	D8537
8-Arm PEG-vinylsulfone, 40 kDa (PEG-VS)	NOF Corporation	N/A
Peptides for PEG-VS functionalization: Glutamine-containing peptide NQEQVSPLERCG-NH2 and lysine-containing peptide with MMP-sensitive sequence AcFKGGGPQGIWGQ-ERCG-NH2. Requirement for peptide synthesis: Purity of >95% and peptide content >80%.	GL Biochem	N/A
Triethanolamine	Sigma	90278
TG-RGD peptide: H-NQEQVSPL-RGDSPG-NH2. Requirement for peptide synthesis: Purity of >95% and peptide content >80%	GL Biochem	N/A
Fibrogammin	CSL Behring	P1250
Thrombin	Sigma-Aldrich	T1063
CaCl_2_	Sigma-Aldrich	C1016
Tris(hydroxymethyl)aminomethane	Sigma-Aldrich	252859
Dextran, 60 000-90 000 kDa	MP Biomedicals	205195
Dimethyl sulfoxide, anhydrous	Sigma-Aldrich	276855-100ML
Glycidyl methacrylate	Sigma-Aldrich	779342-100ML
4-(Dimethylamino)pyridine	Sigma-Aldrich	39405-50G
2-Propanol	VWR	20842.330
Cell adhesive peptide CGRGDS (provided as HCl salt at >95% purity, custom synthesized)	GenScript	N/A
Matrix metalloproteinase (MMP) liable crosslinker peptide CGPQGIAGQGCR (provided as HCl salt at >95% purity, custom synthesized)	GenScript	N/A
Sodium hydroxide (NaOH)	Sigma-Aldrich	S5881-500G
Gelatin from bovine skin	Sigma-Aldrich	9000-70-8
Critical commercial assays		
Polydimethylsiloxane (PDMS) Silicone Elastomer Kit, 10:1 base to curing agent ratio	Dow Corning	Sylgard 184
Experimental models: Cell lines		
bEnd5-td-Tomato cells	Govindasamy et al. 2021^1^	N/A
Experimental models: Organisms/strains		
Mouse_B6C3F1 (wild type females, minimum 6 weeks of age)	Bred in house	N/A
Mouse_Lifeact-GFP (transgenic males, minimum 8 weeks of age)	Riedlet al., 2008^7^	N/A
Other		
SnakeSkin dialysis tubing, 10,000 MWCO	Thermo Fisher Scientific	68100
50 mL conical tubes	Greiner Bio-One	227261
Spin-X centrifuge tube filter, 0.22 μm pore size	Corning	8160
Microcentrifuge	Eppendorf	5424
Centrifuge (rotor A-4-44)	Eppendorf	5804R
Safe-lock 0.5 mL tubes	NeoLAB	296921023
Syringe filter, 0.2 μm pore size	Sa rstedt	831826001
Syringes (1 mL)	Braun inject	9166017v
Needles (30G)	Sterican	TZ-1438
Fine needles (32G)	TSK Laboratory	PRE-32013
Scissors	FST	91460–11
Fine forceps	FST	11254–20
Spring scissors	FST	91500–09
Rubber tubing	Sigma-Aldrich	A5177-5EA
Bunsen burner	Hanau	Touch-O-Matic
Tissue culture dishes (35 mm)	Corning	430588
4-Well dishes	Thermo Fisher Scientific	176740
μ-slide 3D plates	ibidi	81501
Stereo microscope	Zeiss	Discovery V8
CO2 incubator	Sanyo	MCO-5AC(UV)
BenchRocker 2D variable speed rocker	Benchmark Scientific	BR2000
Oven	VWR	4663510
Plasma cleaner with 13.56 MHz generator	Diener Electronic	Femto
Forceps	VWR	232–0209
Acupuncture needles (400 μm diameter)	Hwato	151123
Microscope cover glasses, 24 × 24 mm, No. 1	VWR	631–1571
ThermoMixer C	Eppendorf	N/A
Tissue culture dishes, 100 mm	Sa rstedt	83.3902
Vacuum grease	VWR	DOWC1597418

## Materials And Equipment

### Materials

#### Superovulation

- **PMSG** Dissolve the PMSG powder in 5 mL of the buffer provided by the manufacturer to obtain 200 U/mL of PMSG solution. Add 5 mL of PBS to obtain a final concentration of 100 U/mL. The solution can be stored at −20°C for up to 1 month.
- **HCG** Dissolve the HCG powder in 5 mL of the buffer provided by the manufacturer to obtain 300 U/mL of HCG solution. Add 10 mL of PBS to obtain a final concentration of 100 U/mL. The solution can be stored at −20°C for up to 1 month.

#### Embryo culture medium

- **β-estradiol** Prepare a 10 μM stock solution by dissolving the powder in DMSO. Prepare 50 μL aliquots and store them at −20°C for up to 2 months. Avoid repetitive freeze-thawing.
- **Progesterone** Prepare a 1 mg/mLstock solution by dissolving the powder in DMSO. Prepare 50 μL aliquots and store them at −20°C for up to 2 months. Avoid repetitive freeze-thawing.
- **NAC** Prepare a 50 mM stock solution by dissolving the powder in double-distilled sterile H_2_O. Prepare 100 μL aliquots and store them at −20°Cfor up to 2 months. Avoid repetitive freeze-thawing.
- **FCS** Heat-inactive the FCS at 56°C for 30 min in a water bath. The inactivated serum can be stored at 4°C for 2 weeks or at −20°C for up to 6 months.
- **IVC1 medium** IVC1 medium has been previously described.^8–10^ Supplement DMEM F-12 with 20% heat-inactivated FCS, Pen (25 units/mL)/Strep (25 μg/mL), 2 mM L-Glutamine, 1 × ITS-X, 8 nM β-estradiol, 200 ng/mL progesterone and 25 μM NAC. The medium can be stored at 4°C for 1 week or at −20°C for up to 3 months.

**Table:** 

IVC2 medium			
Component	Stock concentration	Final concentration	Amount
DMEM F-12	100%	77%	77 mL
FCS	100%	20%	20 mL
Penicillin/Streptomycin	100× (5 000 units/mL Pen and 50 mg/mL Step)	0.5× (25 units/mL Pen and 25 μg/mL Strep)	0.5 mL
L-Glutamine	200 mM	2 mM	1 mL
ITS-X	100× (supplier stock)	1×	1 mL
β-estradiol	10 μM	0.8 nM	8 μL
Progesterone	1 mg/mL	200 ng/mL	20 μL
NAC	50 mM	25 μM	50 μL
**Total**			**100 mL**
- **IVC2 medium** For embryo culture in DexMA hydrogels, the IVC2 medium was modified to reduce the amount of sodium bicarbonate, which destabilizes the hydrogel. Dissolve DMEM high glucose powder (without sodium bicarbonate) in ultrapure water and adjust the pH to 7.4. Supplement the DMEM solution with 1.0 g/L NaHCO_3_, 5% heat-inactivated FCS, 30% (vol/vol) KSR, 1 × ITS-X, 8 nM β-estradiol, 200 ng/mL progesterone and 25 μM NAC. The medium can be stored at 4°C for 1 week or at −20°C for up to 3 months.

The low sodium bicarbonate IVC2 medium can be used also for culturing embryos in PEG hydrogels. “Conventional” IVC2 medium can be used as well for PEG hydrogels. This medium has been previously described^8–10^ and slightly modified here as follows. Supplement DMEM F-12 with 5% heat-inactivated FCS, 30% (vol/vol) KSR, Pen (25 units/mL)/Strep (25 μg/mL), 2 mM L-Glutamine, 1 × ITS-X, 8 nM β-estradiol, 200 ng/mL progesterone and 25 μM NAC. The medium can be stored at 4°C for 1 week or at −20°C for up to 3 months.

**Table:** 

IVC2 medium			
Component	Stock concentration	Final concentration	Amount
DMEM F-12 (or) DMEM solution with 1.0 g/L NaHCO3	100%	62%	62 mL
FCS	100%	5%	5 mL
KSR	100%	30%	30 mL
Penicillin/Streptomycin	100× (5 000 units/mL Pen and 50 mg/mL Step)	0.5× (25 units/mL Pen and 25 μg/mL Strep)	0.5 mL
L-Glutamine	200 mM	2 mM	1 mL
ITS-X	100× (supplier stock)	1×	1 mL
β-estradiol	10 μM	0.8 nM	8 μL
Progesterone	1 mg/mL	200 ng/mL	20 μL
NAC	50 mM	25 μM	50 μL
**Total**			**100 mL**

#### bEnd5 cells

- **bEnd5 cell culture medium** Supplement DMEM high glucose with 15% heat-inactivated FCS, Penicillin (50 units/mL)/Streptomycin (50 mg/mL) (Pen/Strep), 2 mM L-Glutamine, 1 mM sodium pyruvate, 1 × NEAA, 0.15 mM β-mercaptoethanol. The medium can be stored at 4°Cfor up to 3 months.

**Table:** 

bEnd5 medium			
Component	Stock concentration	Final concentration	Amount
DMEM high glucose	100%	81%	81 mL
FBS Superior	100%	15%	15 mL
Penicillin/Streptomycin	100× (5 000 units/mL Pen and 50 mg/mL Step)	1 × (50 units/mL Pen and 50 μg/mL Strep)	1 mL
L-Glutamine	200 mM	2 mM	1 mL
Sodium Pyruvate	100 mM	1 mM	1 mL
NEAA	100× (supplier stock)	1×	1 mL
β-mercaptoethanol	14.3 M	0.15 mM	0.7 μL
**Total**			**100 mL**
- **Culture of bEnd5 cells** Coat a 100 mm tissue culture dish with 5 mL0.4% gelatine solution by incubating the plate for 20 min in a humidified incubator at 37°C, 5% CO_2_. Warm the medium and the trypsin at 37°C in a water bath. Thaw a vial of cells from liquid nitrogen storage and resuspend 1 × 10^6^ cells in 5 mL of bEnd5 medium. Pellet the cells by centrifugation at 180 g for 5 min, remove the supernatant and resuspend the pellet in 20 mL of bEnd5 medium. Culture the cells for 3 days, changing the medium every other day. To passage the cells, remove the medium and wash the plate once with PBS. Add 5 mL of 0.25% trypsin to the plate and incubate the cells for 3 min at 37°C, 5% CO_2_. After that, add an equal volume of bEnd5 medium to neutralize the trypsin and pellet the cells by centrifugation at 180 g for 5 min. Resuspend the pellet in bEnd5 medium and replate the cells in a 1:4 ratio. Do not allow the cells to become over 75% confluent, as it may decrease their responsiveness to external stimuli. For co-culturing embryos and endothelial cells, thaw a fresh vial of cells from liquid nitrogen storage. Culture the bEnd5 cells for 3 days and passage the cells once before seeding in the microfluidic device.

#### PEG hydrogel ingredients

- **PEG precursor preparation** Functionalize 8-arm PEG-vinylsulfone, 40 kDa (PEG-VS) (NOF Corporation) with FXIIIa-peptide substrates via Michael-type addition to yield a glutamine-PEG precursor (Q-PEG) and a lysine-PEG precursor (K-PEG) as described in detail in Gjorevski et al.^11^ The amino acid sequence of the glutamine-containing peptide is NQEQVSPLERCG-NH2, and that of the lysine-con-taining peptide with the MMP-sensitive sequence is AcFKGGGPQGIWGQ-ERCG-NH2.
- **TG-RGD** Reconstitute TG-RGD peptide powder to 500 μM concentration stock solution in MilliQ water. Store aliquots of 100 μLor 500 μL at −80°Cfor up to 3 years. Aliquots can be freeze-thawed. Peptide solution is stable over time and has been tested to maintain bioactivity over at least 10 freeze-thaw cycles.
- **10**× **Buffer** Prepare a solution containing 500 mM Tris and 500 mM CaCl_2_. Adjust the pH of the buffer to 7.6. Prepare fresh buffer every 3 months. Store at room temperature. Do not freeze. Long-term storage can alter the pH. It is recommended to make a fresh buffer every 3 months.
- **Fibrogammin** Reconstitute 1250 U of Fibrogammin in 6.25 mL of water provided by the manufacturer. Prepare 1 mL aliquots and store them at −80°C for up to 12 months.
- **Thrombin** Reconstitute thrombin to 20 U/mL final concentration in a buffer containing 10 mM Tris, 150 mM NaCl and 25 mM CaCl_2_,pH7.4. Prepare100 μL aliquots and store them at −80°Cforupto 12 months.
- **Factor XIII (FXIII) activation** Prepare 200 U/mL of activated FXIII by mixing 1 mL of the Fibrogammin solution with 100 μL of the thrombin solution. Incubate the solution at 37°C for 30 min. Prepare 10 μL aliquots and store them at −80°C for up to 12 months. Keep on ice after thawing. Do not refreeze and reuse the activated FXIII. Enzyme activity is reduced after the freeze-thaw cycle, thereby altering the mechanical properties of the gel. Thawed FXIII aliquot should be kept on ice during gel preparation and can be used in this form for 2 h.

#### PEG hydrogel preparation

Mix the ingredients at room temperature in the following order. Add 10% (vol/vol) of the 10× Buffer and 10% (vol/vol) of the RGD peptide to 60% (vol/vol) of water in a 1.5 mL Eppendorf tube. After that, add 10% (vol/vol) of the PEG solution to a final concentration of 1% (vol/vol). Mix the solution by gentle pipetting (pipetting full volume approximately 10 times). Thaw the frozen FXIII stock solution and keep it on ice. Initiate the crosslinking reaction by adding 5% (vol/vol) of the activated FXIII (200 U/mL to a final concentration of 10 U/mL) or, alternatively, dilute the FXIII stock solution (200 U/mL) 1:2 in water and add 10% (vol/vol). Mix the solution vigorously for approximately 1 min by pipetting (pipetting full volume approximately 10 times). The gel should solidify in approximately 5 min after adding the FXIII. An example recipe for the preparation of 90 μL PEG hydrogel is indicated in the table.

**Table:** 

PEG hydrogel		
Reagent	Final concentration	Amount
Water	N/A	54 μL
Buffer	1×	9 μL (10× stock)
PEG	1% (wt/vol)	9 μL (10% wt/vol stock)
RGD	50 μM	9 μL (500 μM stock)
FXIII	10 U/mL	9 μL (100 U/mL stock)
**Total**	**N/A**	**90 μL**

***Note:*** Gelation is initiated approximately 5 min after the addition of FXIII, and gelation is complete after approximately 25 min. An additional 10 μL of gel (i.e., 100 μL total) can be made, which can then be checked for gelation at the bottom of the tube by the pipette tip – gelation is evidenced when the liquid solution forms gel “strings”.

#### DexMA *hydrogel ingredients*

- **Methacrylation of dextran** Synthesis of methacrylated dextran (DexMA) has been previously described.^12^ Dissolve dextran (20 g) and 4-dimethylaminopyridine (2 g) in 100 mL anhydrous dimethyl sulfoxide in a round-bottom flask under vigorous stirring on a magnetic stirrer. Once the solution is clear (this takes a few hours), add 24.6 mL (1.5 M equivalents relative to dextran) glycidyl methacrylate. Transfer the flask to an oil bath on a hot plate and heat the mixture to 45°C for 24 h under continuous stirring. The solution will turn dark brown overnight. Cool the solution to room temperature and add it slowly into 1 L ice-cold 2-propanol to precipitate DexMA. Transfer the precipitated mixture into 50 mL conical tubes and centrifuge at 1600 g for 3 min to collect the solid polymer at the bottom of the tube. Discard the supernatant and dissolve the crude polymer in ultrapure water. Transfer the solution to dialysis tubing and dialyze against ultrapure water for 3 days with two exchanges of solvent daily. This step will eliminate all remaining reagents and side products from the methacrylation procedure and ensure the high purity of DexMA. Finally, lyophilize the solution to obtain DexMA powder. To characterize the degree of methacrylation, perform NMR spectroscopy. For this recipe, a methacrylate/dextran repeat unit ratio of 0.7 is typically obtained.

**Table 5:** 

DexMA synthesis		
Reagent	Final concentration (in dimethyl sulfoxide)	Amount
Dextran	1.2 M (repeat units)	20 g
Dimethyl sulfoxide (anhydrous)	N/A	100 mL
4-Dimethylaminopyridine	164 mM	2g
Glycidyl methacrylate	1.8 M	24.6 mL
**Total**	**N/A**	**158 g**
- **DMEM** Dissolve DMEM powder (without sodium bicarbonate) in ultrapure waterand adjust the pH of the solution to 7.4.
- **DexMA stock solution** Dissolve DexMA in DMEM to a final concentration of 250 mg/mL. Sterilize the solution by passing through a Spin-X centrifuge tube filter (0.22 μm pore size) at a centrifugation speed of 20 000 g for 10 min. Prepare 90 μL or 45 μL aliquots and store them at −80°C for up to 12 months. Once thawed, the aliquot should be used immediately, without any further freezethaw cycles.
- **RGD stock solution** Dissolve the CGRGDS peptide in DMEM (without sodium bicarbonate) at a final concentration of 20 mM. Prepare 90 μL or 60 μL aliquots and store them at −80°C for up to 12 months. After thawing, the aliquots should be used immediately, without further freezethaw cycles.
  ***Note:*** The peptides contain cysteine residues and are therefore prone to oxidation. To avoid the formation of disulfide bonds, the peptides are custom synthesized and stored as hydrochloric acid salts. Therefore, all peptide solutions are acidic, and are only neutralized to initiate the coupling reactions described below.
- **MMP cleavable crosslinker solution** Dissolve the MMP-cleavable crosslinker peptide CGPQGIA GQGCR in DMEM (without sodium bicarbonate) at a final concentration of 200 mg/mL immediately before mixing with the hydrogel precursor solution (see DexMA hydrogel preparation). See the note above on peptide stability.
- **NaOH** Dissolve NaOH in ultrapure water to a final concentration of 1 M and sterilize the solution using a 0.2 μm pore size syringe filter. Prepare 200 μL aliquots and store them at −80°C for up to 24 months. After use, store the leftover solution at 4°C for up to 2 months.

#### DexMA hydrogel preparation

Mix the reagents at room temperature in the following order. Add 27 μL of RGD stock solution (20 mM) in a 0.5 mL Eppendorf tube. Next, add 16 μL of DexMA solution (250 mg/mL), and pipette the solution up and down around 30 times to mix well. To initiate RGD coupling through Michael-type addition, adjust the pH to 8.0 by slowly adding NaOH (1 M) while stirring with the pipette tip. Allow the reaction to proceed for 30 min at room temperature. After that, add 31.4 μL of DMEM. Then, add 11.3 μL of MMP crosslinker solution and mix the solution by pipetting up and down. Immediately centrifuge to remove bubbles and cool the mixture on ice. Once the embryo is ready for hydrogel encapsulation, initiate gelation through Michael-type addition by readjusting the pH to 8.0. Add 65 μL of the precursor solution into the central chamber of the microfluidic device. An example recipe for 90 μL DexMA hydrogel is indicated in the table.

**Table:** 

DexMA hydrogel		
Reagent	Final concentration	Amount
RGD	6 mM	27 μL
DexMA	4.4% (wt/vol)	16 μL
NaOH	see Note	1.15 μL
Incubate 30 min at room temperature		
DMEM	N/A	31.4 μL
MMP Crosslinker	21.8 mM	11.25 μL
NaOH	see Note	3.2 μL
**Total**	**N/A**	**90 μL**

***Note:*** The NaOH volume needed varies slightly (+/-0.2 μL) due to different amounts of hydrochloric acid present in different batches of peptides. For different NaOH volumes, the amount of DMEM needs to be adjusted to achieve a final solution volume of 90 μL.

### Equipment

#### Mouth pipette

Attach to one side of the rubber tubbing a P200 filter tip and on the other side, a P1000 pipette tip without a filter. Pull the glass Pasteur pipette over a flame of the Bunsen burner at an angle of 45° and attach the glass pipette to the P1000 tip as previously shown.^2,13,14^

#### Microfluidic device fabrication

To mimic the structural features of the embryo-maternal interface, apply a microfluidic device, in which pre-formed endothelialized channels are embedded in a 3D DexMA hydrogel. The assembly of the microfluidic device has been previously described.^15^ Follow the individual steps described in detail for a similar device as described previously.^16^ Briefly, generate the master molds by photolithography to fabricate two patterned sheets of PDMS with the required features. Assemble the microfluidic chambers by sealing the PDMS sheets against a glass coverslip using air plasma. Sterilize the devices with UV light for 30 min. To generate channels in the DexMA hydrogel, coat two 400 μm diameter acupuncture needles with a thin layer of gelatin by briefly dipping into a sterile 5% (wt/vol) aqueous solution at 60°C, allow to cool down to 4°C for 3 min to solidify the gelatin solution into a thin hydrogel layer, and insert into the microfluidic device. It is critical to transfer the devices to 4°C immediately to avoid melting the gelatin gel. Continue with the following procedures (DexMA hydrogel fabrication, embryo embedding and endothelial cell seeding) as soon as possible, ideally within a few minutes of needle coating.

#### PDMS-coated dish

Pour 5 g of PDMS precursor solution (10:1 base:crosslinker ratio) into a 10 cm Petri dish. Leave the dish overnight on a flat surface at room temperature. Allow it to solidify further for 4 h in an oven at 60°C.

## Step-By-Step Method Details

### Superovulation

🕓Timing: 48 h

This step describes the hormonal stimulation of the ovary that increases the number of released oocytes available for fertilization.

1 Superovulation.
  - Inject intraperitoneally female mice with 100 μL of PMSG (100 U/mL).
  - After 48 h, administer 100 μLof HCG (100 U/mL) via intraperitoneal injection and set up mating with stud males.
  - Check for a vaginal plug the next day.

### Blastocysts isolation

🕓 Timing: 30 min

This step describes the isolation of E3.5 blastocysts from the uterus.

**Figure 1:**
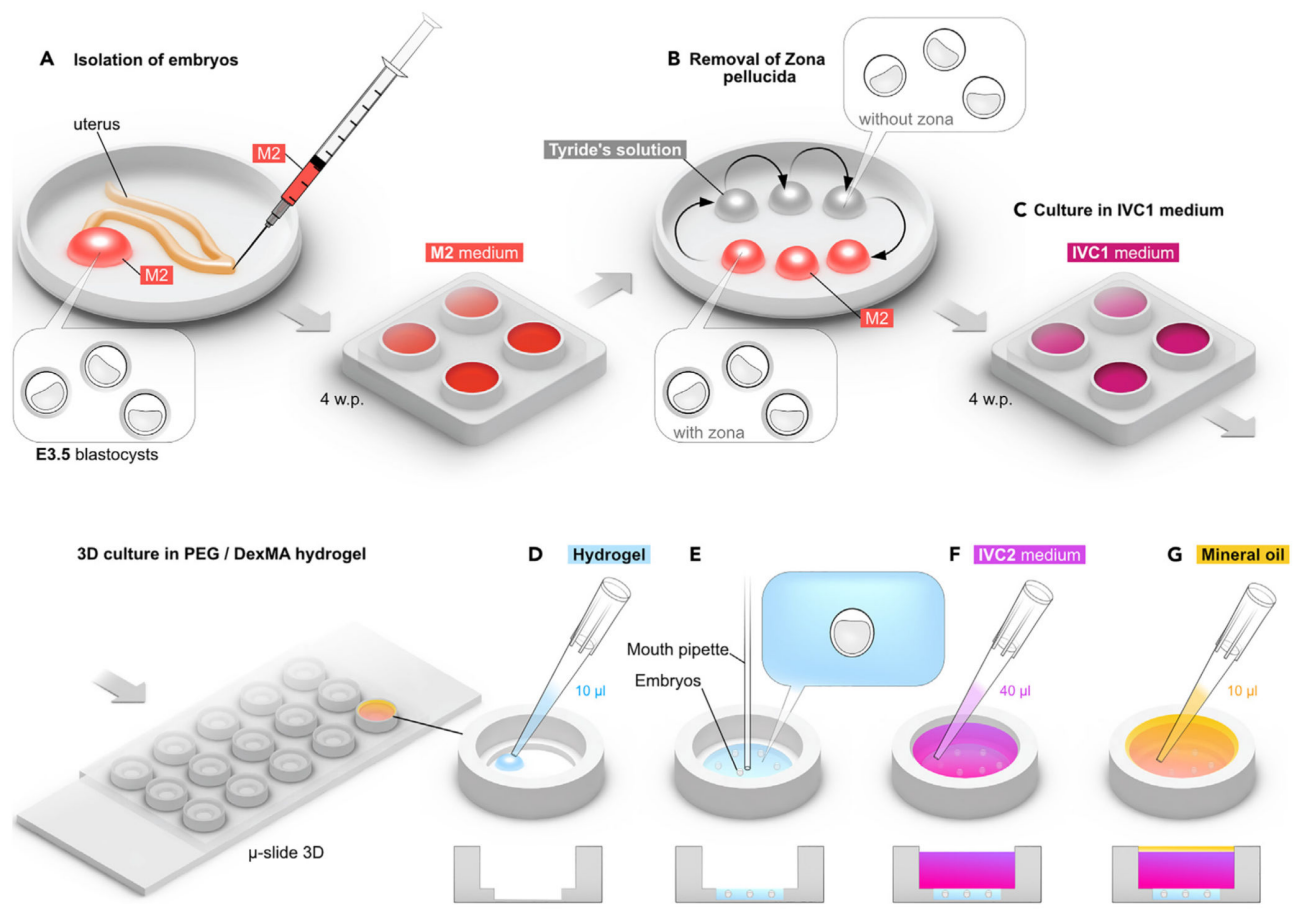
Isolation and culture of mouse embryos in synthetic hydrogels (A-G) Schematic representation of the blastocyst isolation procedure; (A) Isolation of E3.5 blastocysts; (B) Removal of Zona pellucida; (C) Culture in IVC1 medium; (D) Placement of the PEG/DexMA hydrogel into individual wells of the μ-slide 3D plate; (F) Positioning of the embryos into the hydrogel; (F) Adding IVC2 medium to an individual well; (G) Covering the medium with mineral oil to prevent evaporation.

2 Isolation of the uteri.
  - Humanely euthanize the pregnant mice at day three and a half post coitum.
  - Using scissors, cut through the skin and the peritoneum to locate the reproductive tract as previously described.^17,18^
  - Collect the uteri together with the oviduct and the ovaries in a prewarmed M2 medium. Under a stereomicroscope, cut off the ovary and the oviduct using spring scissors.
3 Blastocysts collection.
  - Fill a syringe with a prewarmed M2 medium and attach the needle.
  - Place the needle into the uterine horn and flush the lumen with M2 medium at least 3 times.
  - Flush also the connection between the uterus and the oviduct to ensure that all embryos are released.
  - Collect and transfer the blastocysts into a 4-well platefilled with prewarmed M2 medium using mouth pipetting (Figure 1A). Keep the embryos at 37°C.

### Removal of Zona pellucida

🕓 Timing: 10 min

In this step, the glycoprotein envelope of the embryo is removed via brief exposure to Tyrode’s solution.

**Figure 2:**
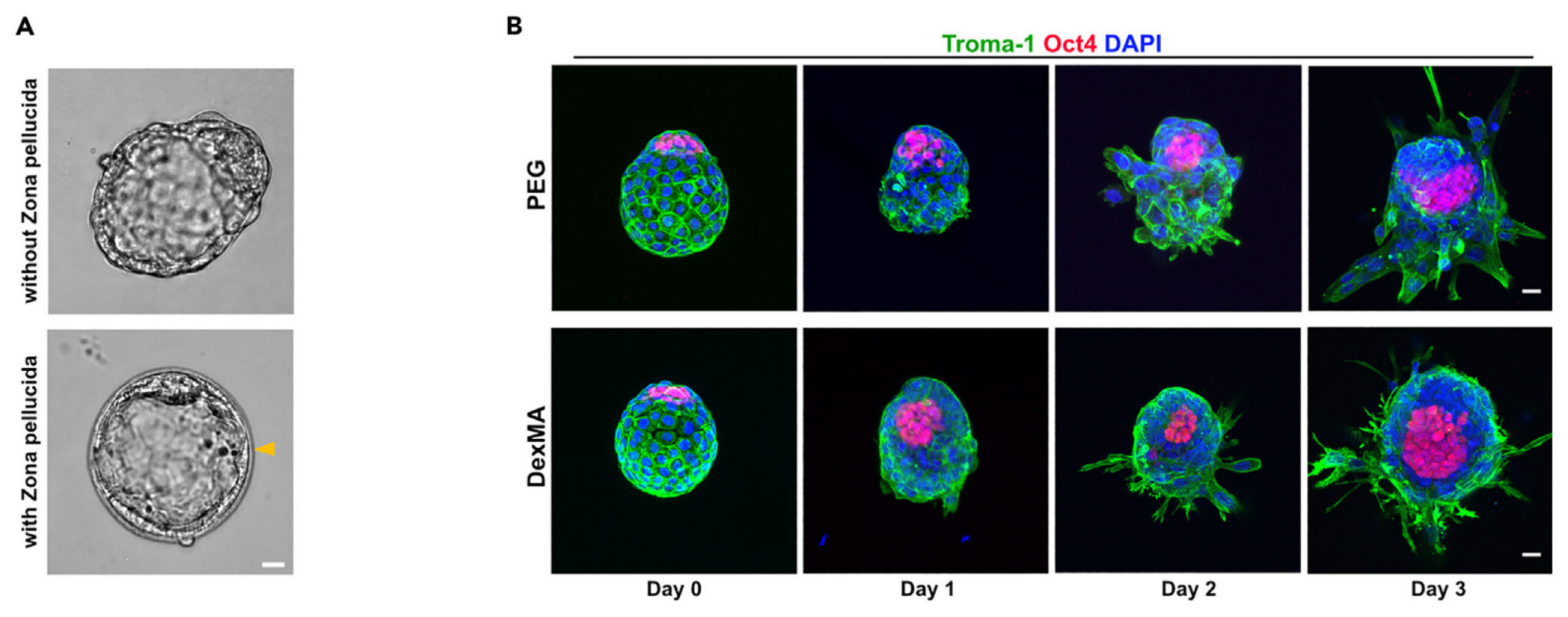
3D culture of mouse embryos in PEG and DexMA hydrogels (A) Blastocyst stage embryos without Zona pellucida (top panel) and with intact Zona pellucida (bottom panel). Arrowhead indicates Zona pellucida. (B) Embryos cultured in PEG or DexMA hydrogels. The embryos were stained for Troma-1, Oct4 and DAPI as previously described.^1^ Z-stacks of images (5 μm step) were acquired using the 10× objective of the Zeiss LSM780 confocalmicroscope system. Scale bars, 20 μm.

4 Tyrode’s solution and M2 medium setup.
  - Invert the lid of a 35 mm dish and place 30 μL drops of prewarmed Tyrode’s solution and 30 μL drops of prewarmed M2 medium.
  - Transfer small groups of 10–20 embryos from the 4-well plate into a drop of M2 medium (Figure 1B).
5 Removal of Zona pellucida via brief exposure to Tyrode’s solution.
  - Remove the remaining medium from the mouth pipette and aspirate Tyrode’s solution. Transfer the embryos into Tyrode’s solution drop.
  - Expunge the remaining liquid and refill the pipette with fresh Tyrode’s solution.
  - Transfer the embryos into the second drop of Tyrode’s solution.
  - Repeat this one more time and observe the quick disappearance of Zona pellucida (within 30 s) in the third drop of Tyrode’s solution (Figures 1B and 2A).
6 Washing embryos in M2 medium.
  - Fill the pipette with medium and immediately transfer the Zona-free blastocysts into a drop of M2 medium.
  - Expunge the remaining liquid and refill the pipette with fresh M2 medium.
  - Repeat this step one more time and transfer the embryos to a 4-well plate filled with prewarmed M2 medium.

⚠ CRITICAL: Zona pellucida has to be removed completely. Observe carefully the embryos, and if a thin layer of Zona pellucida remains, repeat steps 4 to 6. As an alternative approach for Zona pellucida removal, the E3.5 blastocysts can be cultured in an IVC1 medium for 24 h allowing the embryos to hatch naturally. Only the Zone-free embryos can be then used for the 3D culture.

### Embryo culture

🕓 Timing: 3–4 days

This step describes the embryo culture and trophoblast invasion in the synthetic hydrogels.

7 Embryo culture in IVC1 medium.
  - Pipette 1 mL of IVC1 medium into each well of a 4-well plate.
  - Place the plate into a humidified incubator with an atmosphere of 5% CO_2_ in air at 37°C for 30 min for pH and temperature equilibration.
  - Transfer the embryos from the M2 medium to the IVC1 medium (Figure 1C) and culture the blastocysts for 16-20 h in a humidified incubator at 37°C, 5% CO_2_.

⚠ CRITICAL: Do not culture the embryos for more than 24 h in the 4-well plate, as the blastocysts may start to attach to the surface of the plate.

8 Prepare PEG or DexMA hydrogel solution as described in the materials and equipment. Pipette 10 μL of the solution into the inner well of the μ-slide 3D (Figure 1 D).

⚠ CRITICAL: The optical plastic at the bottom of the μ-slide is very thin and prone to cracking due to temperature differences between the slide and the microscope stage. To avoid the formation of cracks, pace the μ-slide in a 10 cm tissue culture plate.

9 Embryo embedding in synthetic hydrogels.
  - Transfer 5–10 blastocysts into the PEG/DexMA hydrogel solution.
  - Place the embryos at an equal distance from each other. Maintain the embryos in the middle of the hydrogel depth by sequential mouth pipetting while the PEG/DexMA solution gradually increases its viscosity.
  - Within approximately 5 min the position of the embryos should remain fixed as the gel polymerizes (Figure 1 E).

⚠ CRITICAL: Transfer of the embryos from the IVC1 medium to the hydrogel solution using as little medium as possible to avoid diluting the hydrogel solution.

⚠ CRITICAL: The blastocysts should be completely embedded in the hydrogel. If the embryos reach the bottom of the plate they will flatten, forming an outgrowth that spreads on the plastic surface. To avoid this the embryos can be placed in the hydrogel at the third minute after pipetting the hydrogel, letting them sink and adjusting their position at the fourth minute.

10 After positioning the embryos, place the μ-slide in a humidified incubator at 37°C, 5% CO_2_ for 15 min (PEG) or at room temperature in a humidified chamber (100 mm Petri dish containing wet paper to the side) for 30 min (DexMA) allowing the hydrogel to polymerize completely.

⚠ CRITICAL: Note: DexMA hydrogel formation through Michael-type addition may occur very fast due to the high efficiency of the reaction. If more time for embryo positioning is required, cooling the precursor solution prior to NaOH addition slows down the process.

11 Pipette 40 μL of IVC2 medium to the upper chamber of the well (Figure 1 F) and add 10 μL mineral oil to prevent evaporation of the medium (Figure 1G). Culture the embryos in a humidified incubator at 37°C, 5% CO_2_.

⚠ CRITICAL: Before adding the IVC2 medium to the μ-slide, equilibrate the temperature and pH of the IVC2 medium at 37°C, 5% CO_2_ for 30 min.

12 Observe the embryo development. By the end of day 1, the mural region of the TE should thicken, and on day 2 the trophoblast should start invading the surrounding environment (Figure 2B).

### Co-culture of embryos and endothelial cells in a microfluidic device

The 3D biomimetic platform also supports the co-culture of embryos and somatic cells, thus providing a model system for studying embryo-maternal interactions. As an example, in steps 13–27 of the protocol, we describe a setup for co-culturing embryos and endothelial cells.

### Superovulation, blastocyst isolation and removal of Zona pellucida

🕓 Timing: 48 h for the superovulation procedure, 30 min for the isolation of the E3.5 blastocysts and 10 min for the removal of Zona pellucida.

13 Isolate blastocysts from superovulated females and remove Zona pellucida following steps 1-6.

### Embryo positioning and culture

🕓Timing: 16–20 h for embryo culture in IVC1,45 min for positioning embryos in the hydrogel and 16 h culture in IVC2 medium.

In this step, the embryos are positioned in the synthetic hydrogel inside of a microfluidic chip.

14 Following the instructions of step 7, culture the embryos in IVC1 medium for 16–20 h in a 4-well plate.
15 Prepare the DexMA hydrogel following the instructions in the materials and equipment. Assemble the microfluidic device as described in the materials and equipment.

⚠ CRITICAL: Once the device and the gelatin-coated needles are put together, the microfluidic chip should be stored at 4°C to prevent the melting of the gelatin coating.

**Figure 3:**
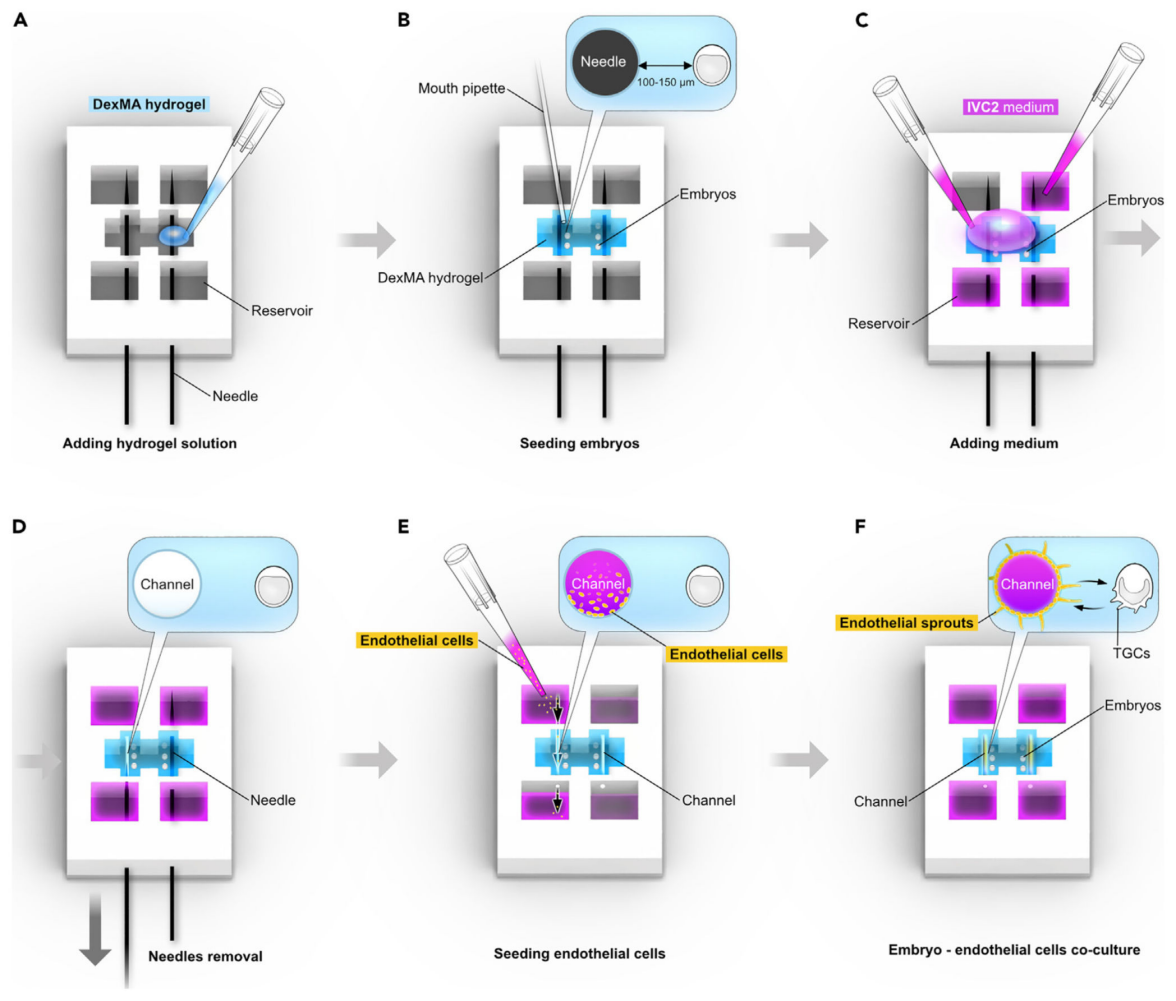
Schematic representation of the co-culture setup of embryos and endothelial cells in the microfluidic device (A–F) (A) Placement of the DexMA hydrogel into the microfluidic device; (B) Positioning of the embryos in close proximity to the channels; (C) Filling the reservoirs with IVC2 medium; (D) Removal of the needles; (E) Seeding of the endothelial cells; (F) Embryo – endothelial cells co-culture.

16 Embryo seeding in the reservoir containing DexMA hydrogel.
  - Pipette 65 μL of the DexMA hydrogel solution into the middle reservoir of the device (Figure 3A).
  - Following step 9 of the main PROCEDURE, transfer 5–7 embryos into the hydrogel. Position the embryos in close proximity of 100-150 μm to the channel.
  - Maintain the position of the embryos for approximately 5 min by mouth pipetting, while the hydrogel polymerizes (Figure 3B).

⚠ CRITICAL: As the hydrogel solidifies quickly, collect the embryos from the IVC1 medium in the mouth pipette, just before adding NaOH to the hydrogel solution that initiates polymerization.

⚠ CRITICAL: Place the embryos in close proximity to the channel (100–150 μm) so the trophoblast and the endothelial cells are not too far apart to interact.

**Figure 4:**
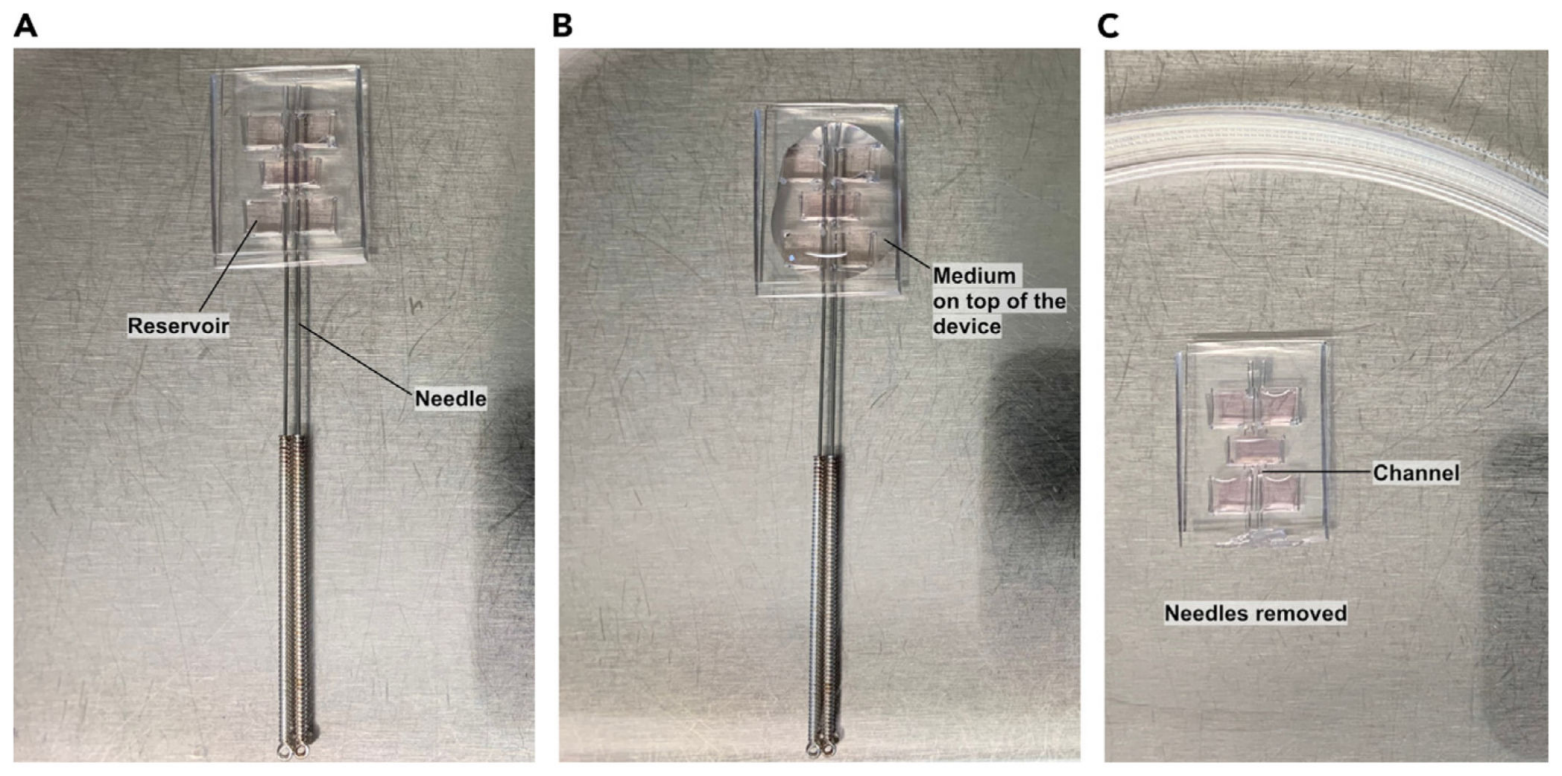
Microfluidic device (A-C) Photographs of the microfluidic device before (A and B) and after (C) removal of the needles.

17 After positioning the embryos:
  - Place the device in a humidified chamber (100 mm Petri dish containing wet paper to the side) at 37°C, 5% CO2 for 30 min allowing the hydrogel to polymerize completely.
  - Add 500 μL of IVC2 medium on top of the device covering the hydrogel chamber and the 4 reservoirs (Figures 3C, 4A, and 4B).
  - Place the device (in a 100 mm Petri dish containing wet paper to the side) in a humidified incubator at 37°C, 5% CO2 and incubate overnight.

### Seeding of endothelial cells and co-culture with mouse embryos

🕓Timing: approximately 5–6 h for the seeding of endothelial cells and approximately 3 days for the embryo-endothelial cell co-culture.

This step describes the seeding of the endothelial cells in the channels of the microfluidic device for co-culture with the embryos.

18 Place the device in a tissue culture hood and carefully pull the needles out using forceps (Figures 3D and 4A-4C).

⚠ CRITICAL: Pull the needles out slowly using forceps. While pulling, hold the device using another forceps and keep the pulling direction straight to prevent damaging the channels by the tip of the needle.

⚠ CRITICAL: Before pulling the needles out, ensure that the reservoirs of the device are filled with IVC2 medium and that there is also some medium on top of the device. As the needles are pulled out, the medium flows from the reservoirs into the channels. At the same time, the reservoirs are refilled by medium on top of the device.

19 Seal the open ends where the needles were inserted using vacuum grease to prevent medium leakage from the reservoirs.
20 Removal of the excess medium.
  - Carefully remove any excess medium from the surface of the sealed device.
  - Using filter paper, absorb any traces of medium from the bottom surface of the device.
  - Place the device on a 100 mm plate coated with PDMS (see materials and equipment) and ensure that the device has well adhered to the PDMS.
21 Washing of the channels.
  - Wash the channels, as hydrogel or gelatin debris can cause clogging.
  - For each channel, aspirate most of the medium from the channel and the reservoirs. Add fresh medium to one of the reservoirs and let the medium flow through the channel to the reservoir on the other side.
  - Repeat this procedure one more time.

⚠ CRITICAL: Do not remove the medium completely from the reservoirs, as this may deplete the fluid in the channel causing the channel surfaces to stick and close.

22 Prepare bEnd5 cell culture as described in the materials and equipment. Dissociate the cells using trypsin and resuspend the pellet in IVC2 medium at a concentration of 10 × 10^6^ cells/mL.

⚠ CRITICAL: Using this specific concentration of cells is important to prevent over- or underpopulation of the channels with endothelial cells.

23 Seeding of the endothelial cells.
  - Aspirate approximately 3/4 of the medium from the reservoirs and pipette 80 μL of the endothelial cell suspension into one of the reservoirs of each channel (Figure 3E).
  - Observe the endothelial cells flowing through the channels into the opposing reservoir to monitor the cell seeding. Once the cells pass through the channel, add 80 μL of cell suspension into each reservoir.
  - Incubate the device at 37° C, 5% CO_2_ for 90 min. The cells will settle to the bottom half of the channel wall by gravity.
24 Aspirate approximately 3/4 of the endothelial cell suspension from the reservoirs and add fresh cell suspension, repeating step 11. However, this time invert the dish with the attached device so that the cells settle by gravity and adhere to the opposite half of the channel wall during the 90 min incubation.
25 Washing the channels containing endothelial cells.
  - Remove approximately 3/4 of the medium containing the endothelial cells from the reservoirs and add fresh IVC2 medium.
  - Incubate the device for 30min at 37°C, 5% CO_2_. The endothelial cells that are not in the channels settle to the bottom of the reservoirs.
  - After the 30 min incubation, scrape off the layer of endothelial cells in the reservoirs using a P10 pipette tip.
  - Remove the medium containing the scraped cells.
  - Add fresh medium to the reservoirs and let the medium flow through the channels to wash unattached endothelial cells. Repeat this one more time to remove any unattached endothelial cells from the channels and the reservoirs.

⚠ CRITICAL: Do not remove the medium completely from the reservoirs, as this may deplete the fluid in the channels causing them to close.

26 Add fresh IVC2 medium to the reservoirs and place the PDMS-coated plate containing the device on a rocker inside a cell culture incubator (BenchRocker, 30° tilt angle and 12 rpm rocking speed). Incubate the device on the rocker at 37°C, 5% CO_2_ overnight and add fresh medium in the morning.

⚠ CRITICAL: Place wet paper on the side of the Petri dish to prevent medium evaporation.

**Figure 5:**
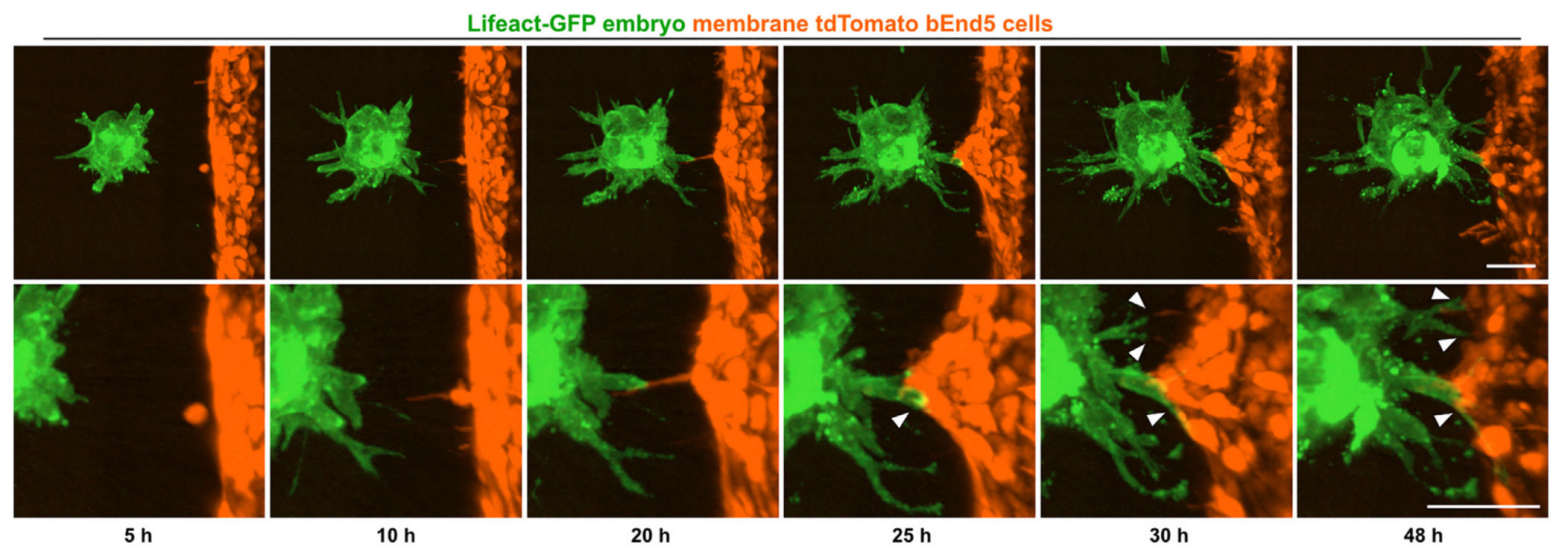
Co-culture of embryos and endothelial cells in the microfluidic device Snapshot images of time-lapse microscopy of Lifeact-GFP embryo and tdTomato-expressing endothelial cells co-cultured in the microfluidic device. Arrowheads indicated cell-cell contacts between the trophoblast and the endothelial cells. The imaging was performed using an Andor Dragonfly spinning disc confocal microscope equipped with an sCMOS camera using 525 nm and 620 nm excitation lasers with 2%–5% laser power. The images were captured every 1 h, with an optical slice distance of 1.5 μm, using 10× CFI P-Apo Lambda objective. Scale bars, 100 μm.

27 Embryo–endothelial cells co-culture.
  - On the next day, the endothelial cells and the embryos can be co-cultured in an incubator at 37°C, 5% CO_2_ for the next 3 days, and the medium should be exchanged every 36 h.
  - Alternatively, the device can be placed on a heated microscope stage, inside an environmental chamber at 37°C, 5% CO_2_ for live imaging.
  - The first interactions between the invasive trophoblast and the endothelial cell sprouts can be observed in the next 15–36 h (Figures 3F and 5).

## Expected Outcomes

During the first 16–20 h culture in the IVC1 medium, the blastocyst cavity should expand After transfer in the hydrogel, the mural TE region should thicken by the end of day 1 of the 3D culture, which is indicative of the initiation of TE differentiation to TGCs. On day 2, the trophoblast should start invading the surrounding environment (Figure 1C). Virtually all embryos should develop invasive trophoblast. The TGCs should downregulate TE markers, such as Cdx2, activate the expression of TGCs markers, such as Hand1, and gain the expression of vascular genes, such as VE-cadherin, Pe-cam1, Dll4, Vegf and Pdgf receptors.^1^ The same sequence of events is expected when the embryos are co-cultured with endothelial cells. In the microfluidic device, the trophoblast cells in close proximity to the channels should form direct cell-cell contacts with the endothelial sprouts (Figure 2B).

## Limitations

The maternal environment consists of multiple cell types forming specialized tissues that support the development of the early embryo. The biomimetic platform, which is a reductionist approximation of the stromal environment, lacks such complexity. In contrast to the relatively quick trophoblast invasion *in vivo* (E4.5 – E5.5), embryos cultured *in vitro* exhibit a delay, which can be attributed to a potential adaptation period to the culture conditions and/or suboptimal signaling cues, normally provided by the maternal environment.

The hydrogels are designed to support trophoblast invasion and co-culture with somatic cells mimicking embryo-maternal interactions in the stromal compartment of the decidua. However, the functionality of the somatic tissues is limited compared to their *in vivo* counterparts. For instance, the embryo can establish direct cell-cell contacts with endothelial sprouts ex utero, but the “artificial” blood vessels are not linked to functional circulation that can support long-term embryogenesis *in vitro*.

## Troubleshooting

### Problem 1

No embryos are found after flushing the uterus (related to Step 3).

### Potential solution

If no embryos are found, use freshly prepared solutions of HCG and PMSG. Use young mice of approximately 6 weeks of age derived from hybrid strains (e.g., F1 generation of C57Bl/6 and C3H). The hybrid strains tend to be more responsive to hormonal stimulation. The male mice need at least 2 days of rest between matings.

### Problem 2

Inefficient removal of Zona pellucida (related to Step 6).

### Potential solution

If Zona pellucida remains intact or it takes more than 2–3 min to disappear in the third drop of Tyrode’s solution, this indicates that traces of M2 medium are present in the solution, neutralizing its activity. Refill the pipette with Tyrode’s solution and transfer the embryos to a fresh drop of Tyrode’s solution.

### Problem 3

Hydrogel gelation takes a long time (related to Step 10).

### Potential solution

If the PEG hydrogel does not polymerize in 5 min or in a maximum of 7 min for DexMA, check the shelf life of the buffers, the quality of FXIII (PEG) or the concentration of NaOH (DexMA).

### Problem 4

The trophoblast cells do not invade the surrounding environment (related to Step 12).

### Potential solution

If the embryos do not invade the hydrogel, there might be a thin layer of Zona pellucida remaining. If the embryos are Zona-free but do not develop invasive trophoblast, check the shelf life of the IVC1 and IVC2 media and the hydrogel components, especially the RGD.

### Problem 5

The embryos and the channels in the microfluidic device are difficult to observe (related to Step 16).

### Potential solution

To improve the visibility of the channel and the embryos, while positioning the blastocysts, use gooseneck lights in addition to the illuminated stage of the stereomicroscope.

### Problem 6

DexMA hydrogel gelation takes a long time (related to Step 17).

### Potential solution

If the hydrogel does not polymerize in 5 min, this might be because the microfluidic device has been stored at 4°C and it is still cold.

### Problem 7

Clogging of the channels (related to Step 21).

### Potential solution

If a channel is clogged, add medium in one of the reservoirs and tilt the device slightly, allowing the medium to flow to the reservoir on the other side. Avoid generating air bubbles at the channel opening, as they can block the entry of the medium to the channel. If air bubbles are formed, gently remove them using a pipette tip.

### Problem 8

The endothelial cells do not attach to the channel (related to Step 24).

### Potential solution

If the endothelial cells do not attach to the wall of the channels, this could be due to prolonged tryp-sinization or subconfluent cell culture. Thaw a fresh vial of cells and follow closely the instructions for bEnd5 cell culture in the materials and equipment.

### Problem 9

The channels are not well covered by the endothelial cells (related to Step 24).

### Potential solution

If the channels are scarcely populated with endothelial cells, repeat steps 11 and 12 and increase the incubation times by 30 min.

### Problem 10

The endothelial cells do not form angiogenic sprouts (related to Step 27).

### Potential solution

If the endothelial cells do not form sprouts, thaw a fresh vial of bEnd5 cells and passage the cells once before seeding them into the channels.

## Resource Availability

### Lead contact

Further information and requests for resources and reagents should be directed to and will be fulfilled by the lead contact, Ivan Bedzhov (ivan.bedzhov@mpi-muenster.mpg.de).

### Materials availability

- This study did not generate new unique reagents.

## Data Availability

This study did not generate datasets and code.

## Highlights

3D biomimetic environment harboring biomechanical properties of the uterine stroma

Trophoblast invasion of embryos cultured in synthetic hydrogels

Co-culture of embryos and somatic cells in a microfluidic device

A platform for modeling the cellular interactions at the embryo-maternal interface
